# Association between ventricular CSF biomarkers and outcome after shunt surgery in idiopathic normal pressure hydrocephalus

**DOI:** 10.1186/s12987-023-00475-8

**Published:** 2023-10-25

**Authors:** Rebecca Grønning, Anna Jeppsson, Per Hellström, Katarina Laurell, Dan Farahmand, Henrik Zetterberg, Kaj Blennow, Carsten Wikkelsø, Mats Tullberg

**Affiliations:** 1 Hydrocephalus Research Unit, Department of Clinical Neuroscience, Institute of Neuroscience and Physiology, Sahlgrenska Academy, University of Gothenburg, Blå Stråket 7, Sahlgrenska University Hospital, 41345 Gothenburg, Sweden; 2 Department of Medical Sciences, Neurology, Uppsala University, Uppsala, Sweden; 3 Clinical Neurochemistry Laboratory, Sahlgrenska University Hospital, Mölndal, Sweden; 4 Department of Psychiatry and Neurochemistry, Institute of Neuroscience and Physiology, Sahlgrenska Academy, University of Gothenburg, Mölndal, Sweden; 5 Department of Neurodegenerative Disease, UCL Institute of Neurology, Queen Square, London, UK; 6 UK Dementia Research Institute at UCL, London, UK; 7 Institute at UCL, London, UK; 8 Hong Kong Center for Neurodegenerative Diseases, Clear Water Bay, Hong Kong, China; 9 Wisconsin Alzheimer’s Disease Research Center, University of Wisconsin School of Medicine and Public Health, University of Wisconsin-Madison, Madison, WI USA

**Keywords:** Normal pressure hydrocephalus, Cerebrospinal fluid, Prediction, Neurodegeneration, Amyloid, Ventricular catheter, Peritoneal catheter

## Abstract

**Introduction:**

The relationship between neurochemical changes and outcome after shunt surgery in idiopathic normal pressure hydrocephalus (iNPH), a treatable dementia and gait disorder, is unclear. We used baseline ventricular CSF to explore associations to outcome, after shunting, of biomarkers selected to reflect a range of pathophysiological processes.

**Methods:**

In 119 consecutive patients with iNPH, the iNPH scale was used before and after shunt surgery to quantify outcome. Ventricular CSF was collected perioperatively and analyzed for biomarkers of astrogliosis, axonal, amyloid and tau pathology, and synaptic dysfunction: glial fibrillary acidic protein (GFAP), chitinase-3-like protein 1 (YKL40/CHI3L1), monocyte chemoattractant protein-1 (MCP-1) neurofilament light (NfL), amyloid beta 38 (Aβ38), Aβ40, Aβ42, amyloid beta 42/40 ratio (Aβ42/40), soluble amyloid precursor protein alfa (sAPPα), sAPPβ, total tau (T-tau), phosphorylated tau (P-tau), growth-associated protein 43 (GAP43), and neurogranin.

**Results:**

The neurogranin concentration was higher in improved (68%) compared to unimproved patients (median 365 ng/L (IQR 186–544) vs 330 (205–456); *p* = 0.046). A linear regression model controlled for age, sex and vascular risk factors including neurogranin, T-tau, and GFAP, resulted in adjusted R^2^ = 0.06, *p* = 0.047. The Aβ42/40 ratio was bimodally distributed across all samples, as well as in the subgroups of improved and unimproved patients but did not contribute to outcome prediction. The preoperative MMSE score was lower within the low Aβ ratio group (median 25, IQR 23–28) compared to the high subgroup (26, 24–29) (*p* = 0.028). The T-Tau x Aβ40/42 ratio and P-tau x Aβ40/42 ratio did not contribute to shunt response prediction. The prevalence of vascular risk factors did not affect shunt response.

**Discussion:**

A higher preoperative ventricular CSF level of neurogranin, which is a postsynaptic marker, may signal a favorable postoperative outcome. Concentrations of a panel of ventricular CSF biomarkers explained only 6% of the variability in outcome. Evidence of amyloid or tau pathology did not affect the outcome.

## Introduction

Among the dementia disorders, only a few are considered reversible, idiopathic normal pressure hydrocephalus (iNPH) probably being the most important. In addition to cognitive decline, iNPH patients suffer from impairment of gait and balance, and urinary incontinence, and show characteristic enlargement of the ventricles with disturbed CSF dynamics [1]. Shunt surgery is effective in up to 80% of patients and should be performed without delay [2, 3]. Swedish population-based studies have suggested that 2% of people above 65 years of age and 5.9–8.9% of people aged over 80 may suffer from iNPH, whereas probably only 20–40% of patients are diagnosed and treated [4, 5]. One reason for this is the current lack of simple and reliable markers for diagnosis and prediction of outcome. Such markers should reflect fundamental pathophysiological processes of the iNPH state suggested to appear in periventricular brain regions [6].

In spite of the often striking reversibility of symptoms after surgical treatment, the pathophysiology of iNPH and the effect of the CSF dynamic disturbance on brain function remains largely unknown [7]. CSF and brain extracellular fluid have a close interchange of molecules, suggesting that the contents of the CSF could mirror the metabolic events within the brain parenchyma [8]. Lumbar CSF and blood biomarkers are increasingly used for diagnostic and prognostic purposes in neurological disorders, e.g. Alzheimer’s disease (AD), thus having an explanatory value for understanding cell physiology, in addition to diagnosis of neurological conditions [9]. In iNPH, reduction of CSF concentrations of amyloid precursor derived proteins, total (T-tau), and phosphorylated tau (P-tau), in addition to increased neurofilament light (NfL), and monocyte chemoattractant factor 1 (MCP-1) have been reported which, together with reduced periventricular perfusion, indicate a reduced periventricular metabolism and axonal degeneration [10]. The combination of total tau, Aβ40, and MCP-1 has been shown to have a high diagnostic discriminability for iNPH in relation to clinical mimics, with an AUC of > 0.8 but the value of these markers for prediction of shunt response needs to be investigated [11]. No lumbar CSF biomarker has yet proved able to aid in the decision on whether to shunt or not to shunt.

Vascular disease and AD are among the known comorbidities of iNPH. In AD, the typical lumbar CSF biomarker pattern comprises reduced Aβ42 and a low Aβ42/40 ratio, increased T-tau, P-tau and neurogranin, compared to controls [9, 12]. Kazui et al. found that an increased T-tau/Aβ42 as a measure of AD pathology was associated with poorer shunt response in iNPH patients whereas other studies reported no negative influence of AD pathology on outcome [13]. Also vascular risk factors and subcortical small vessel disease are prevalent in iNPH [7].

Analysis of biomarkers in ventricular CSF could probably prove more sensitive to changes in brain metabolism than in lumbar CSF, ventricular CSF being in close proximity to the periventricular brain regions of interest in iNPH. Jeppsson et al. recently reported a postoperative increase of ventricular CSF NfL, APP derived proteins, and P-tau, whereas levels of T-tau decreased [11].

Our aim was to study the predictive value of biomarkers of neuronal degeneration (neurofilament light (NfL) for axonal white matter damage; total tau (T-tau) for general neurodegeneration), Alzheimer’s disease tau pathology (phosphorylated tau (P-tau)), astrogliosis (monocyte chemoattractant protein 1 (MCP-1), glial fibrillary acidic protein (GFAP), and chitinase-3-like-protein (C3LP1/YKL40)), and proteins of the amyloid cascade (soluble amyloid precursor protein alfa and beta (sAPP–α, sAPPβ), amyloid beta 40 (Aβ40), Aβ42, and Aβ42/40 ratio) in ventricular CSF in order to study a wide spectrum of metabolic events in close proximity to areas of interest in the brain. We hypothesized that synaptic dysfunction could be involved due to the reversibility of symptoms, which is a possible sign of synaptic plasticity, in iNPH, and therefore we included two representative markers previously studied in neurodegenerative disorders as candidate markers for synaptic dysfunction: GAP43 (a presynaptic biomarker) and neurogranin (a postsynaptic biomarker) [14, 15].

A secondary aim was to explore CSF biomarker evidence of coexisting Alzheimer’s disease and its predictive value.

## Methods

### Study design and participants

Patients diagnosed with iNPH who were subjected to shunt surgery and had a postoperative follow-up at median 5 months were consecutively included in the Gothenburg POiNT study, conducted between 2014 and 2017 at two sites, Sahlgrenska University Hospital, Gothenburg and Östersund hospital, Östersund [16, 17]. Of the 143 included patients from the original study group, 24 patients lacked peroperative ventricular CSF sampling and were thus excluded, leaving a total of 119 participants (Gothenburg n = 110 and Östersund n = 9). All patients received a ventriculo-peritoneal (n = 115), or a ventriculo-atrial (n = 4) shunt (PS Medical strata; Medtronic). At follow-up, all patients’ shunts were examined for patency by evaluation of clinical symptoms and CT or MRI. If doubts regarding shunt patency remained following CT or MRI, a radionuclide shuntography or a lumbar infusion test was performed [18].

### Outcomes

Clinical symptoms were assessed pre- and post-operatively on the iNPH scale introduced by Hellström et al., comprising domains of gait, balance, cognition and urinary incontinence [17]. Outcome was defined as postoperative score minus preoperative score (delta iNPH scale score). A postoperative increase in the iNPH scale score of five points or more defined clinical improvement [17].

### Procedures

Eight ml of ventricular CSF was collected during the surgical intervention, immediately after shunt insertion and a discard of the first 2 ml. All CSF analyses were performed at the Neurochemistry Laboratory at Sahlgrenska University Hospital, by board-certified laboratory technicians who were blinded to clinical data. Aβ-related biomarkers (Aβ40, Aβ42, sAPPα and sAPPβ) and MCP1 were analyzed by electrochemiluminescence assays (Meso Scale Discovery, Rockville, MD, USA). Validated in-house ELISA methodology was used to analyze NfL [19), neurogranin [15), GAP43 [20) and GFAP [21), whereas CSF levels of T-tau, and P-tau were measured using commercially available Lumipulse technology (Fujirebio, Ghent, Belgium), as previously described [22]. YKL-40 was measured using Human Chitinase 3-like 1 Quantikine ELISA Kit (R&D Systems, Minneapolis, MN) [22]. All concentrations are given in ng/L. All samples were analyzed in one round of experiments using one batch of reagents by board-certified laboratory technicians who were blinded to clinical data. Intra-assay coefficients of variation, monitored using internal quality control samples in the beginning and end of each run, were below 10%.

### Statistical analysis

Patients with a biomarker value outside 3 SD from the mean were considered outliers and excluded: 3 GFAP outlier values and 1 NfL outlier value were excluded. Total iNPH scale score was normally distributed whereas biomarker concentrations were all skewed. The cut-off value for Aβ42/40 ratio between patients of high and low ratio respectively, was judged by eyeball-test appreciation. Ventricular CSF biomarker concentrations could be reported for a range of 115 to 119 patients. Distributions were checked for normality. All variables not considered normally distributed were log10-transformed before being entered into linear regression analysis. Aβ42/40 ratio had a bimodal distribution, it was thus not included in the regression analysis.

The tau/Aβ42 ratio was calculated in accordance with Kazui et al., $(P or T)Tau\times A\upbeta 40 \div A\upbeta 42$ [13]. For correlations, the tau/Aβ42 ratio was transformed by log10. CSF marker levels were compared across groups of improved and unimproved patients using Mann Whitney U test. Student’s t-test was used for comparisons as applicable. Uni- and multivariate linear regression models were built with delta iNPH as the dependent variable. Biomarkers with an alpha value of < 0.1 in a univariate linear regression were included in multivariate regression models. In a hypothesis-based approach, the diagnostic biomarker combination of MCP-1, T-tau and Aβ40 reported by Jeppson et al. was included in a separate multivariate regression model [23]. Adjustments were made for age, sex and vascular risk factors. A p-value of < 0.05 was considered statistically significant. No correction for multiple comparisons was conducted. SPSS version 29.0.0.0 (IBM SPSS Statistics) was used in all statistical analyses.

### Ethical considerations

This study was performed in accordance with the Declaration of Helsinki and was approved by the Regional Ethical Review Board in Gothenburg (Dnr 328–14, T439-15). All patients or their next of kin gave written consent to inclusion in the study.

## Results

The mean age of the 119 patients was 74 (± 7) (± SD), and 43 (36%) were female. Sixty-eight percent of the patients improved after surgery (Table 1). The average time from diagnosis to surgical intervention was 106 ± 52 days. No significant differences were seen between improved and not improved patients with regard to age, sex, BMI, vascular risk factors (history of heart disease, hypertension, diabetes mellitus), sleep duration, duration of symptoms, time from diagnosis to surgery, and MMSE score. A missing case analysis showed an increased delay to surgery for the 24 excluded patients, 140 ± 77 days compared to 106 ± 52 (*p* = 0.009) but no additional differences could be shown for the variables in Table 1.

**Table 1:** Demographic data of 119 iNPH patients

	All patients	Unimproved	Improved	P
	n = 119	n = 38 (32%)	n = 81 (68%)	
Duration of symptoms (months)	36 (21–48)	36 (23–49)	30 (10–41)	0.995 *
Time from diagnosis to surgery (days)	106 ± 52	108 ± 9	105 ± 6	0.817§
Age (years)	74 ± 7	76 ± 7	74 ± 7	0.930§
Sex (men)	n = 76 (64%)	n = 27 (71%)	n = 49 (60%)	0.310 ‡
Body mass index	27 ± 4	27 ± 5	27 ± 3	0.224§
Vascular risk factors: heart disease, hypertension, or Diabetes mellitus (yes)	n = 84 (71%)	n = 28 (74%)	n = 56 (69%)	0.671 ‡
Heart disease (yes)	n = 35 (29%)	n = 13 (34%)	n = 22 (27%)	0.282‡
Hypertension (yes)	n = 70 (59%)	n = 23 (61%)	n = 47 (58%)	0.478‡
Diabetes mellitus (yes)	n = 36 (30%)	n = 11 (29%)	n = 25 (31%)	0.505‡
MMSE	26 (24–28)	26 (21–31)	26 (22–30)	0.630 §
Preoperative iNPH scale score	54 ± 17	54 ± 17	53 ± 17	0.759§
Postoperative iNPH scale score	66 ± 18	56 ± 17	70 ± 17	< 0.001§
Delta iNPH scale score	12 ± 11	2 ± 2	17 ± 9	< 0.001§

Values are given as mean (± SD) or median (IQR), MMSE = mini mental state examination. A delta iNPH scale score < 5 defines unimproved patients, while a delta iNPH scale score of ≥ 5 indicates improved patients. P-values represent comparisons across groups of unimproved and improved patients

*Mann Whitney U test

§Student’s t-test

‡*Chi*^2^ test.

Biomarker concentrations are shown in Table 2.

**Table 2:** Biomarker levels in peroperative ventricular CSF of iNPH patients

	N	Mean (± SD) (ng/L)	Median (IQR) (min–max) (ng/L)
Aβ40	119	4256 (± 2151)	3880 (2620–5570) (914–11,740)
Aβ42	119	346 (± 189)	314 (193–428) (68–988)
Aβ42/40	119	0.08 (± 0.02)	0.09 (0.07–0.1) (0.04–0.12)
APPα	119	90 (± 58)	73 (52–123) (18–321)
APPβ	119	228 (± 122)	203 (144–295) (31–673)
P-tau	119	63 (± 54)	49 (34–74) (14–377)
T-tau	119	834 (± 891)	617 (373–1030) (75–7110)
NfL	118	1102 (± 754)	845 (655–1243) (160–4980)
GFAP	116	1297 (± 1208)	935 (64–1420) (200–6660)
YKL40	117	107 (± 48)	99 (71–135) (34–307)
MCP1	117	479 (± 166)	440 (364–543) (151–1140)
Neurogranin	117	434 (± 279)	351 (245–566) (74–1700)
GAP43	115	2625 (± 1556)	2233 (1532–3107) (540–8463)

Apart from neurogranin, there were no significant differences between the groups (Table 3).

**Table 3:** Peroperative ventricular CSF biomarker concentrations of subsequently unimproved and improved patients

	Unimproved (n = 38) Median (IQR) (ng/L)	Improved (n = 81) Median (IQR) (ng/L)	*P* ‡
Aβ40	3900 (2486–5314)	3840 (2210–5470)	0.752
Aβ42	316 (197–436)	312 (197–435)	0.462
Aβ42/40	0.08 (0.065–0.095)	0.09 (0.075–0.105)	0.080
APPα	79 (43–115)	68 (37–100)	0.433
APPβ	218 (134–303)	203 (128–279)	0.745
P-tau	45 (28–62)	50 (30–70)	0.162
T-tau	523 (298–749)	637 (294–981)	0.059
NfL	870 (601–1139)	820 (530–1110)	0.470
GFAP	960 (566–1354)	900 (505–1295)	0.650
YKL40	99 (74–125)	99 (66–133)	0.871
MCP1	458 (380–536)	437 (350–524)	0.535
Neurogranin	330 (205–456)	365 (186–544)	0.046
GAP43	2323 (1507–3139)	2167 (1365–2970)	0.257

‡ Mann Whitney U test

In the univariate linear regression analyses only GFAP, T-tau, and neurogranin met the requirements for inclusion in a multivariate regression model (*p* < 0.1) (Table 4).

**Table 4:** Univariate linear regression analyses of biomarkers

	Pearson r	Unstandardized B	95% CI	Adjusted R^2^	*P* ‡
Aβ40	− 0.063	− 2.780	− 7.028 to 50.844	− 0.005	0.497
Aβ42	0.021	0.880	− 6.698 to 8.457	− 0.008	0.819
APPα	− 0.148	− 5.192	− 11.554 to 1.170	0.014	0.109
APPβ	− 0.086	− 4.184	− 12.285 to 3.917	− 0.001	0.351
P-tau	0.132	5.062	− 1.945 to 12.069	0.009	0.155
T-tau	0.169	5.246	− 0.361—10.853	0.020	0.066
NfL	− 0.112	− 4.609	− 12.146 to 2.928	0.004	0.228
GFAP	− 0.160	− 5.514	− 11.837 0.809	0.017	0.087
YKL40	− 0.061	− 3.190	− 12.857 to 6.476	− 0.005	0.515
MCP1	− 0.124	− 8.979	− 22.273 to 4.316	0.007	0.184
Neurogranin	0.181	7.095	− 0.029 to 14.218	0.024	0.051
GAP43	0.021	0.873	− 6.823 to 8.569	0.008	0.823

Ventricular CSF biomarkers as independent variables, transformed by log10, and delta iNPH scale score after shunt surgery as a dependent variable. ‡ By Pearson correlation

Age correlated inversely on trend level with the delta iNPH scale score: Pearson R -0.180, unstandardized B -0.272 95% CI − 0.544 to 0.000 Adjusted R^2^ 0.017, p = 0.050.

Multiple regression analysis with ventricular CSF biomarkers GFAP, T-tau, and neurogranin as independent variables showed an Adjusted R^2^ of 0.043 and p-value of 0.051. When adjusted for potential confounding factors of age, sex and presence of vascular risk factors, a multivariate model resulted in an adjusted R^2^ of 0.061, and a p-value of 0.047, (Table 5).

**Table 5:** Multivariate linear regression analysis on n = 114 patients with ventricular CSF biomarkers showed an adjusted R^2^ of 0.061 and a statistical significance of *p* = 0.047

	Pearson R	Unstandardized β coefficients	95% CI	*p‡*
Constant		35.108	0.134 to 70.082	0.049
GFAP	− 0.158	− 7.002	− 13.743 to − 0.260	0.042
T-tau	0.161	4.772	− 14.262 to 23.806	0.620
Neurogranin	0.171	1.067	− 22.890 to 25.024	0.930
Age	− 0.184	− 220	− 0.493 to 0.053	0.114
Sex	− 0.154	3.137	− 7.028 to 0.755	0.113
Vascular risk factors	− 0.035	0.187	− 3.976 to 4.349	0.929

Biomarkers transformed by log10, and age, sex, and vascular risk factors as independent variables, and outcome on the iNPH scale score after shunt surgery as a dependent variable. Biomarkers selected had an alpha value of < 0.1 in the univariate regression. *‡ By Pearson correlation*

**Fig. 1:**
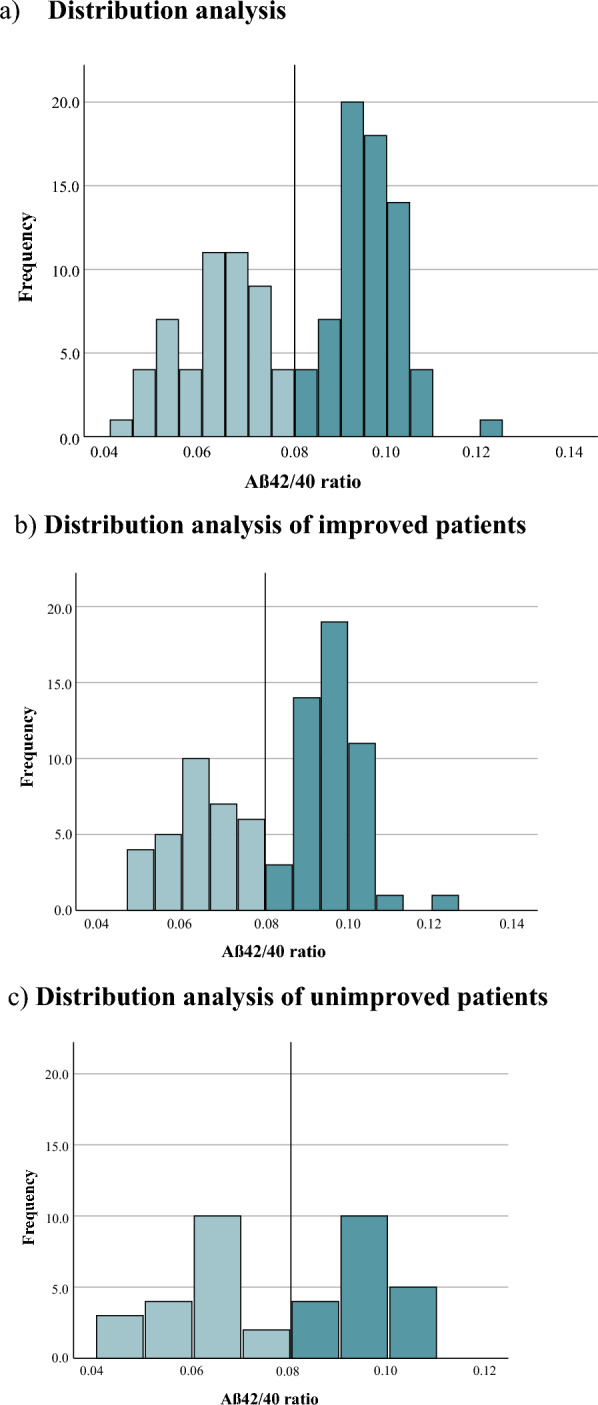
**a** Distribution of ventricular CSF Aβ42/40 ratio in all patients with a cut-off of 0.08.** b** Distribution within subgroup of improved patients. **c** Distribution for unimproved patients. Cut-off remains at ~ 0.08

The hypothesis-based combination of T-tau, Aβ40, and MCP-1 was included in a multivariate linear regression which was not statistically significant when considering its correlation to clinical outcome (*p* = 0.107). When neurogranin was added to the model, the correlation was still not statistically significant (*p* = 0.155) (Fig. 1).

### Alzheimer’s disease biomarkers: the amyloid β 42/40 ratio

**Fig. 2:**
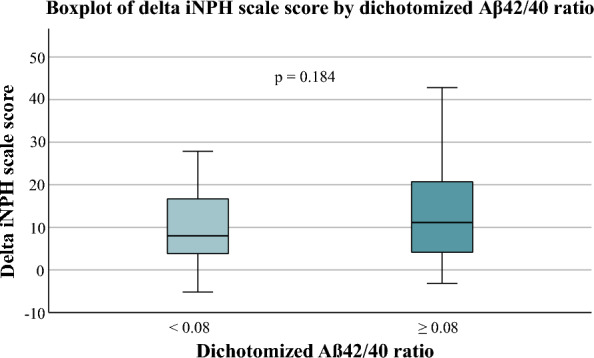
Boxplot of the delta iNPH scale score in patients with a low Aβ42/40 ratio (< 0.08) (n = 51) and a high ratio (≥ 0.08) (n = 68). No difference was noted between the groups (*p* = 0.184)

**Fig. 3:**
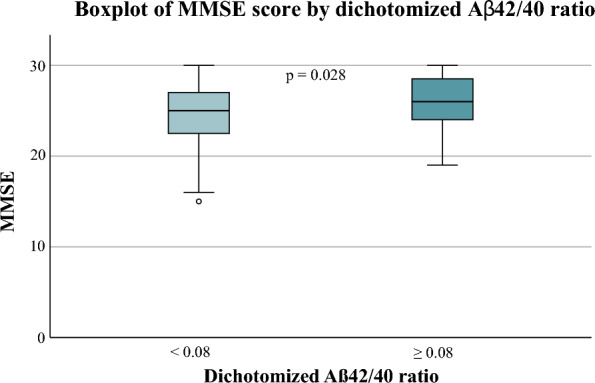
Comparison of MMSE scores between patients with a low amyloid ratio and a high amyloid ratio (*p* = 0.028,* Mann Whitney U test*)

The distribution of amyloid ratio (Aβ42/40) was bimodal, with a low amyloid ratio (n = 51, 43%) and a high amyloid ratio (n = 68, 57%) respectively with a cut-off of 0.08, with similar patterns in improved and unimproved patients (Fig. 2a–c). There was no difference in the delta iNPH scale score between iNPH patients with a high amyloid ratio and those with a low amyloid ratio (Fig. 3).

The baseline scores for MMSE were reduced for patients with a low amyloid ratio (median 25, IQR 23–28) compared to the high subgroup (median 26, IQR 24–29) (*p* = 0.028). Levels of neurogranin were equal for low 346 (median 346 ng/L, IQR 203–490) and high (median 356 ng/L, IQR 153–560) Aβ ratio groups (*p* = 0.699).

### Alzheimer’s disease biomarkers: tau proteins

AD-specific biomarkers were analyzed between the amyloid pathology groups. Levels of T-tau did not differ between low Aβ ratio (median 668 ng/L, IQR 439–897) and high 545 (545 ng/L, IQR 163–927) (*p* = *0.982*). The levels of P-tau also did not differ between low Aβ ratio (median 58 ng/L, IQR 43–73) and high (45 ng/L IQR 23–66) (*p* = 0.200).

A construction reported by Kazui et al. of the T-tau x Aβ40/Aβ42 ratio was not correlated to outcome (*p* = 0.741), unstandardized B 1.215 ± 3.663 (− 6.054 to 8.484 95% CI), adjusted R2 − 0.009 [13]. A construction of the P-tau x Aβ40/Aβ42 ratio was not correlated to outcome (*p* = 0.247), unstandardized B 0.002 ± 0.002 (− 0.001 to 0.005 95% CI), adjusted R2 0.003. The tau-ratios were equally distributed between improved and unimproved patients (T-tau x Aβ40/Aβ42 *p* = *0.918*, P-tau x Aβ40/Aβ42 *p* = 0.419).

## Discussion

Previous studies on iNPH pathophysiology have shown disturbance of CSF dynamics, dysmetabolism, astrogliosis, and injuries, predominantly to subcortical regions [6]. The current use of CSF biomarkers in research has focused on elucidating the pathophysiological mechanisms as well as aiding diagnosis, so far without clear conclusions [24]. No robust biomarker for prediction of outcome after shunt surgery is available. The biomarker profile in ventricular CSF of iNPH patients and its relation to outcome is unknown. We studied markers of astrocyte activation, subcortical neuronal damage, proteins of the amyloid cascade, and AD markers as well as synaptic proteins in a large group of iNPH patients and explored their associations to clinical outcome after shunt surgery to elucidate pathophysiological mechanisms related to shunt response in NPH.

We found a relative increase in the synaptic protein neurogranin to be associated with postoperative improvement. A positive correlation of a magnitude that did not reach statistical significance, but sufficient to grant inclusion in a regression model, was found. Neurogranin is a postsynaptic protein previously suggested to be specific for AD reflecting synaptic plasticity [9]. The increased concentration in improved patients, albeit with a significant overlap, indicates that synaptic function may be involved in the reversibility of iNPH, revealing a novel aspect of iNPH pathophysiology. GAP43, a presynaptic protein involved in memory function and information storage and the other synaptic biomarker analyzed here [25], was not related to postoperative outcome which, taken together with the neurogranin findings, hypothetically could suggest that changes on the postsynaptic rather than the presynaptic region are involved in the reversibility of iNPH. As additional markers for synaptic dysfunction have recently been investigated by Nilsson et al., inclusion of these novel markers could be beneficial for future investigations of synaptic function in iNPH [26].

The multivariate regression model including the three markers GFAP, neurogranin and, T-tau showed a weak correlation to outcome, explaining only 6% of the variance. This finding implies that other mechanisms not accounted for here are the key determinants for prediction of outcome and that the pathophysiological phenomena of astrogliosis, synaptic dysfunction and subcortical neuronal degeneration, signaled by changes in biomarker concentrations, play a minor role in moderating shunt response in patients. In the multivariate model, adjustment for potential confounders of age, sex and vascular risk factors resulted in a slightly stronger correlation, which we interpret to mean that these characteristics can influence the pathophysiology. As for patient demographic factors, age was weakly negatively correlated with outcome. A hypothesis model of diagnostic iNPH markers T-tau, Aβ40, and MCP-1 was not predictive of shunt response, indicating that they merely reflect a diagnostic fingerprint of the disorder not related to outcome.

The influence of comorbidities such as AD for outcome in iNPH patients is important to consider. Here we report a bimodal distribution of the Aβ42/40 ratio in the ventricular CSF of iNPH patients. The bimodular distribution of Aβ ratio suggests two patient groups: one with evidence of amyloid pathology and one without. Regardless of evidence of amyloid pathology, the response to shunting was equal. We conclude that evidence of amyloid pathology should not exclude patients from iNPH investigation or shunting, a notion supported by others [27]. We believe that a subgroup of iNPH patients suffer from comorbid AD rather than amyloid plaque depositions as a part of the iNPH pathology. Amyloid plaques are found among 30–60% of iNPH patients [28]. A study of AD biomarkers in ventricular CSF and subsequent brain pathology at autopsy could aid in the understanding of differences in pathogenesis between these diseases.

The prevalence of amyloid deposits in the brain of iNPH patients has previously been suggested to affect treatment effect, increase levels of T-tau, or reduce Aβ42 [29]. However, in lumbar CSF, described by Lukkarinen et al., levels of P- and T-tau were not increased among patients with amyloid depositions peroperatively which our results based on ventricular CSF confirm [28]. In contradiction to Kazui et al., here the T-tau/Aβ42 ratio did not affect outcome, even if the more AD-specific marker P-tau was used instead of T-tau. The lower MMSE scores in patients with a low Aβ ratio suggest a more prominent cognitive defect, adding evidence to the postulation of comorbid AD. However, Migliorati et al. described increased levels of T-Tau and P-Tau to correlate with poor shunt response, not seen in our material [30]. Although in other studies, increased levels of Tau and high levels of Aβ38, 40 and 42 has mainly been found to correlate poorly with cognitive function, a clinical outcome measure not investigated here [31, 32]. Furthermore, an increase of the marker neurogranin has been suggested to predict future neurodegeneration in Alzheimer’s and serves as an early mechanism-of-action marker to aid in diagnosis of rapid disease [12]. Our finding of higher concentrations among improved patients may indicate that neurogranin is in fact not specific to AD, but rather a sign of unspecific postsynaptic activation. Another possible explanation of higher neurogranin concentrations, may be presence of comorbid prodromal AD. However, comorbid AD is not expected to be associated with a better outcome, so we find this explanation unlikely. Levels of neurogranin in Aβ 42/40 ratio subgroups do not aid further in the postulation if the patients suffer from AD since neurogranin could be increased regardless of whether amyloid pathology exists or not among AD patients [33]. Here, neurogranin was equal, regardless of evidence of amyloid deposition. It could be valuable to look more deeply into the symptomatology and pathophysiological mechanisms of the Aβ ratio subgroups separately.

Vascular comorbidities are known to be frequent in iNPH [34]. Hypertension, heart disease and diabetes mellitus were not correlated to shunt response, either separately or together. NfL, a biomarker which has been shown to increase among patient groups with prominent vascular pathophysiology such as subcortical small vessel disease, did not affect shunt response in our material [34]. A study on vascular comorbidities is required to elucidate the role of such comorbidities further, with a suggestion to also include additional vascular comorbidities and diffusion and perfusion MRI examination of subcortical areas.

The validated clinical outcome scale used here can be considered to give an objective measure of outcome, analyzed by specialists with experience and knowledge of iNPH diagnostics. CSF samples were analyzed by trained laboratory technicians using established methods. With a study sample of 119 patients diagnosed according to international criteria, we therefore believe that our data can be considered robust and representative however in need of replication. No comparison of concentration differences in ventricular CSF between patients and healthy controls was possible mainly for ethical reasons, resulting in difficulty with interpretation. Amyloid cascade proteins are lower in the lumbar CSF of iNPH patients in comparison to AD, which has been interpreted as amyloid mis-metabolism and not AD-specific amyloid pathology in iNPH [35]. Levels have been found altered to be compared to healthy individuals as well. In a future project, other markers such as the Leucine-rich α-2-glycoprotein, a biomarker suggested for prediction of iNPH diagnosis, although not yet validated for prediction of shunt response, could be included in the analysis [36]. Additional comorbidities, as well as the other markers for synaptic function, not assessed here could potentially affect outcome and could also be included in future studies for improved understanding. We did not include a measure of ventricular size such as Evan’s index in our analyses. It cannot be ruled out that differences in ventricular size may influence biomarker concentrations, e.g. by a dilution effect. However, in an earlier study including some of the biomarkers analyzed here, we concluded that these biomarkers were not influenced by CSF volumes [37]. Future studies should address this question. We chose not to perform corrections for multiple comparisons as we consider our study explorative. Our findings should, however, be tested for replication.

In summary, a relative increase in the synaptic marker neurogranin was seen among iNPH shunt responders, although with a significant overlap between groups. Biomarkers reflecting astrogliosis, neuronal axonal degeneration and synaptic dysfunction were all associated with the magnitude of improvement, but weakly. Patients with signs of amyloid pathology improved to the same extent as those without. The role of vascular risk factors in iNPH remains to be further investigated.

## Data Availability

We support data sharing within the restrictions of the ethical approval. Data will be shared upon reasonable request. Requests can be made to the corresponding author.
